# All in This Together? A Preregistered Report on Deservingness of Government Aid During the COVID-19 Pandemic

**DOI:** 10.1017/XPS.2021.10

**Published:** 2021-03-31

**Authors:** Aengus Bridgman, Eric Merkley, Peter John Loewen, Taylor Owen, Derek Ruths

**Affiliations:** 1 McGill University, Montréal, Canada; 2 University of Toronto, Toronto, Canada

**Keywords:** Deservingness, redistribution, COVID-19, Canada, social affinity

## Abstract

The COVID-19 pandemic has placed unprecedented pressure on governments to engage in widespread cash transfers directly to citizens to help mitigate economic losses. Major and near-universal redistribution efforts have been deployed, but there is remarkably little understanding of where the mass public believes financial support is warranted. Using experimental evidence, we evaluate whether considerations related to deservingness, similarity, and prejudicial attitudes structure support for these transfers. A preregistered experiment found broad, generous, and nondiscriminatory support for direct cash transfers related to COVID-19 in Canada. The second study, accepted as a preregistered report, further probes these dynamics by comparing COVID-19-related outlays with nonemergency ones. We find that COVID-19-related spending was more universal as compared to a more generic cash allocation program. Given that the results were driven by the income of hypothetical recipients, we find broad support for disaster relief that is not means-tested or otherwise constrained by pre-disaster income.

The COVID-19 pandemic has been met with unparalleled government fiscal action and mass public mobilization. While citizens participated in a massive social coordination effort to stop the spread of the coronavirus, governments around the world engaged in large-scale stimulus spending that far outstripped what was spent during, and in the aftermath of, the 2008 financial crisis. A major portion of this spending has been in the form of cash transfers directly to citizens (Gentilini et al. 2020). These spending measures have been implemented at great cost and with a much-needed urgency. And yet, we have limited knowledge of the types of financial transfers with broad public support. Critical to developing this understanding is research into perceptions of deservingness which underline social welfare policy preferences and political decisions (Petersen 2012). Existing literature suggests that discriminatory attitudes, a “hierarchy of deservingness”, and similarity evaluations are likely to drive these policy preferences, but we find that during a society-level crisis such as COVID-19, these normal patterns are laid aside in favor of a more universal and collectivist set of preferences. This universal generosity is particularly strong for COVID-19 financial relief, indicating support for universal redistribution during crisis times.

## Deservingness in times of crisis

Previous research has shown the importance of both subjective and affective deservingness heuristics for complex policy decisions (Skitka and Tetlock 1993). The extant literature identifies three considerations that are particularly important for untangling when and under what conditions support for social spending is higher: deservingness criteria, discriminatory attitudes, and similarity.

First, deservingness evaluations rely on a set of criteria (control, attitude, reciprocity, identity, and need, see Oorschot et al. 2017) that explicitly or implicitly distinguish between those who are seen to be deserving of relief and those who are regarded as undeserving (Oorschot 2000). Characteristics commonly associated with “deservingness” include illness, unemployment, and the presence of children in the household. Jensen and Petersen (2017) find that those who are sick or require healthcare are typically deemed the most deserving. While evaluations of deservingness of the unemployed can vary based on context, those who are un- or underemployed through no fault of their own are typically perceived as more deserving (Oorschot 2006). Finally, those with (young) children are perceived as more deserving of aid relative to their peers without children (Will 1993).

Not all dimensions of deservingness are based on objective considerations, however. Notably, prejudicial attitudes such as those related to ethnicity and race can significantly modify perceptions of deservingness (DeSante 2013; Hjorth 2016). In Canada, the USA, and the United Kingdom, race has been shown to be a powerful determinant of public support for redistribution (although effects are contextual and specific racial minority groups are differently disadvantaged across national contexts, see Harell, Soroka, and Iyengar 2016). Further, citizenship and immigration status are also important factors, with a body of evidence showing that immigrants are generally perceived as the least deserving group for social spending (Ford 2016; Reeskens and Meer 2019). Kootstra (2016) finds these effects in both the United Kingdom and the Netherlands. She finds between a 0.2 and 0.35 standard deviation decrease in perceived deservingness for hypothetical Jamaican and Pakistani profiles, as compared to a hypothetical British one. She also estimates a 0.2 standard deviation penalty for non-native-born profiles. These effects are modest but persistent across countries and ethnic groups.

These prejudicial attitudes may be compounded by well-documented so-called similarity preferences, where support for redistributive policies tends to be higher when the beneficiaries of redistribution are perceived as similar to oneself (Cavaillé and Trump 2015; Fong 2001). Two conceptually distinct motivations are critical to understanding these similarity preferences: social affinity and material self-interest. Social affinity studies find that people care more about the well-being of others with whom they share certain characteristics (Kristov, Lindert, and McClelland 1992); thus, demonstrating that generosity is at least somewhat conditional on one’s ability to empathize with the potential recipient. Furthermore, the literature on material self-interest indicates that decisions around deservingness can be influenced by a desire to capture government resources for one’s own group, community, or other set of interests (Campbell et al. 1960). Differentiating between the two mechanisms is challenging. Nonetheless, objective indicators of similarity have been shown to powerfully predict redistributive attitudes (Chong, Citrin, and Conley 2001). In a society where one group is larger than others, these similarity preferences can compound already strong prejudicial preferences and lead to a wide gap in support for policies that benefit majority and minority populations (Ford 2016).

These three explanatory factors generally describe support for redistribution during “normal” times. However, during a time of crisis, we have strong reason to believe that the strength of these considerations is relaxed and that a more universal approach toward cash transfers will be adopted. This universalism is likely to emerge for instrumental, collectivist, and structural reasons. We describe each in turn.

The first set of considerations that may increase support for universal cash transfers are instrumental ones – allocations may be higher because they serve an important function in reducing the severity of a disaster. In the case of a pandemic, providing a substitute for employment income reduces the number of people leaving their homes for nonessential reasons and thus can limit human-to-human transmission. Universality may also be preferred over means-tested or other more complex program delivery mechanisms to ensure rapid response and enable behavioral change.^1^

Second, greater collectivism may be produced by a common sense of loss, deservingness, and vulnerability. As described above, deservingness is linked to perceptions of victimization, particularly when the need for support is linked to events outside the locus of control of the intended recipient (Skitka and Tetlock 1993). During a pandemic, this subjective evaluation of deservingness is likely higher for all subject populations; existing prejudicial attitudes and motivations of material self-interest may be overridden as everyone is deemed equally deserving. Finally, individuals are likely to perceive themselves and their families as facing high levels of risk. This shared vulnerability may prompt more empathetic and generous attitudes. These collectivist impulses may be especially true when concerns about national well-being are primed (Olivera 2014). Empirical research in this area is limited, with previous work on economic crises yielding mixed results. Some have found that crises such as the Great Recession do not shift attitudes toward redistribution in the aggregate (Brooks and Manza 2013; Soroka and Wlezien 2014), while others find evidence of increased support (Rosset and Pontusson 2014).

The third reason why a more universal approach toward cash transfers may be adopted is structural. The nature of existing government responses may change the way individuals make social spending decisions. A large and generous response may broadly increase support for such measures. The Canadian government has engaged in the largest direct cash transfer program in its history^2^, and a deep literature has repeatedly shown that individuals who reside in institutional environments with more generous and universal welfare states have higher support for nondiscriminatory welfare programs (Laenen 2018; Larsen 2008; Van Der Waal, De Koster, and Van Oorschot 2013). It is likely that the existence of these programs can be linked to greater support for universal cash transfers specifically designed to mitigate personal economic loss due to COVID-19.

Certainly, there appears to be more universalism during the COVID-19 pandemic, with governments around the world facing little criticism for unprecedented, large-scale individual transfers, even those targeted at specific populations that may have been perceived as less deserving in the past. We conducted two studies to evaluate the extent to which the different deservingness, prejudicial, and similarity factors impact preferences during the COVID-19 crisis. The findings from Study 1 informed the hypothesis and design for Study 2, so we first describe the hypotheses tested in Study 1 and provide those results.

## Study 1: Support for COVID-19 cash transfers

A preregistered initial probe into these dynamics consisted of a survey of 2,522 Canadian citizens 18 years and older from the online panel provider Dynata. The survey was fielded from May 21 to 27, 2020. National level quotas were set on region (i.e. Atlantic, Quebec, Ontario, and West), age, gender, and language. Respondents were weighted within each region of Canada by gender and age to match population benchmarks from the 2016 Canadian census.

We employed a paired conjoint design. Each respondent was presented with two pairs of profiles of hypothetical Canadian residents. These profiles consisted of seven randomized features: (1) name (including clear ethnicity/gender indicators); (2) citizenship; (3) health status; (4) marital status; (5) children; (6) employment status; (7) and income before the pandemic. This resulted in 10,088 assessed profiles with 3,072 possible permutations, each of which was equally likely with no constrained permutations. Respondents were then asked the following for each profile: “How much money should **Name** receive from the government during the pandemic per month?, and were given a slider to report their answer from $0–4,000 (slider resting point was at $0)”.^3^ Details on the exact instrument can be found in the Supplementary Materials. Similar instruments have been deployed in other research that examines the deservingness of government allocations (e.g. Harell, Soroka, and Iyengar 2016; Hjorth 2016; Will 1993).^4^

We anticipated the results from Study 1 to align with the preponderance of the literature as described above; allocation of government benefits would follow documented deservingness, prejudicial, and similarity considerations. We expected those profiles with common deservingness characteristics (young children, preexisting health issues, lost income and employment due to the pandemic, and those with lower prepandemic incomes) to be perceived as the most deserving. We also anticipated existing prejudicial attitudes, which would negatively penalize non-Whites and noncitizens.

We moreover anticipated that similarity would shape respondents’ redistribution preferences. First, we expected the effects of income or job loss due to the pandemic on allocation to be higher among those whose job is at risk from the pandemic, or among those who have already been laid off. Second, we anticipated the effect of children on allocation to be higher for those with children. Third, we anticipated the effect of preexisting health conditions on allocation to be higher if they, or someone in their household, has had a recent illness. Finally, we expected the negative effect of pre-pandemic income on allocation to be higher among low-income respondents, and potentially reverse direction for high-income respondents. Details on the similarity variables can be found in the Supplementary Materials.

Results are reported below, with no evidence found for similarity considerations, little evidence for prejudicial attitudes, and mixed evidence of deservingness considerations.

## Study 1 Results

**Figure 1:**
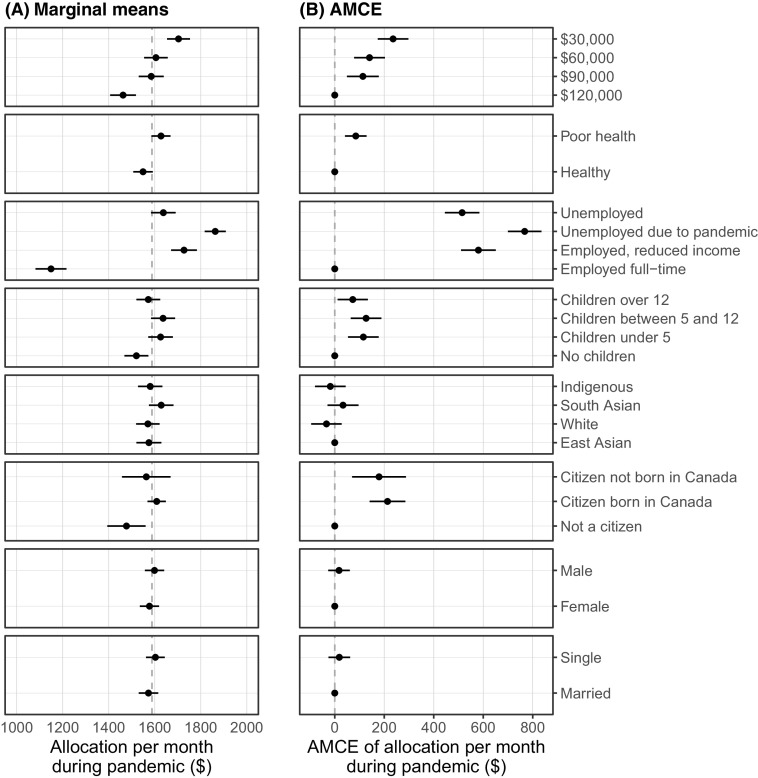
(A) Marginal Means and (B) AMCEs. Dashed Line in (A) is the Grand Mean. *NOTE*: 95% Confidence Intervals are Shown.

Figure 1 provides the results for Study 1. Figure 1(A) illustrates the marginal means that test unconditional expectations, while Figure 1(B) showing the average marginal component effects (AMCEs). The grand mean of the suggested allocation across all assessed profiles was $1,588, indicating respondents were inclined to support a substantial amount of government assistance during the pandemic. Contrary to our preregistered expectations, there were no significant differences between allocation for Whites ($1,570) and non-Whites ($1,593).

Most of our other expectations were met to an unexpectedly small degree. Noncitizens were allocated less assistance ($1,477) than citizens, but there was no difference between those who were ($1,608) or were not born in Canada ($1,563). People with children were allocated more support than people with no children ($1,520), with this particularly true for those with children under 5 ($1,625) and between 5 and 12 ($1,636). People with preexisting conditions were allocated more ($1,627) than those without ($1,549). Pre-pandemic income was also negatively associated with deservingness. People earning $30,000 prior to the crisis were allocated more ($1,703) than those earning $60,000 ($1,605), $90,000 ($1,584), and $120,000 ($1,462).

By far the largest effect we found was attributed to employment status. As expected, people who are employed and unaffected by the pandemic were allocated, on average, far less ($1,149) than those who lost income ($1,727) or employment as a result of the pandemic ($1,862). Those who were unemployed for reasons unrelated to the pandemic were allocated less than both these groups ($1,637).

**Figure 2:**
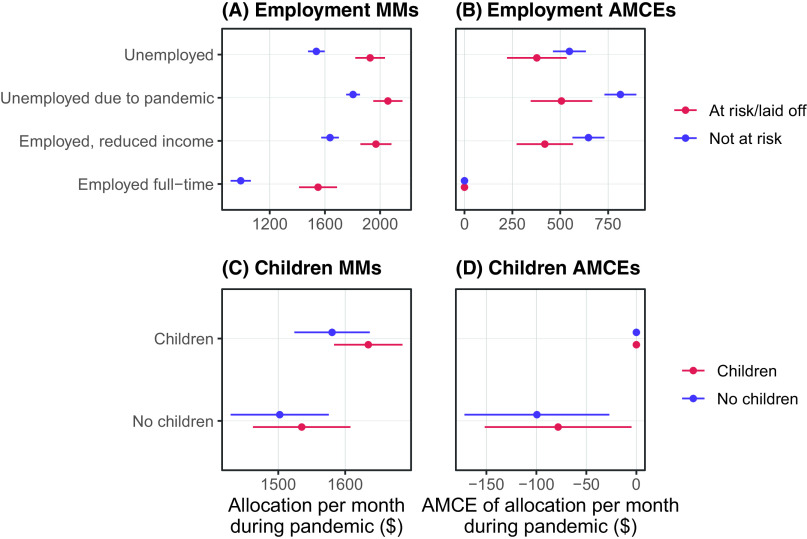
Sub-group marginal means for employment status (A) and children (C). Sub-group AMCEs for employment status (B) and children (D). *NOTE*: 95% Confidence Intervals are Shown.

Figure 2 presents the results of our similarity-based tests for employment status (A and B) and children (C and D) using subgroup AMCEs since we anticipated subgroup differences in causal effects. We also provide side-by-side comparisons with the subgroup marginal means in this figure because the interpretation of AMCEs can be sensitive to the choice of reference category (Leeper, Hobolt, and Tilley 2020). We find little evidence that there are any social affinity or material self-interest dynamics driving allocations. The effects of income and employment loss on allocation amount are similar for those whose employment is at risk (or were laid off) and those whose employment is not in jeopardy, contrary to expectations (Figure 2A). The only significant difference is that there was relatively higher allocations for those unemployed for reasons unrelated to the pandemic among those not at risk of unemployment, which we had not anticipated.

**Figure 3:**
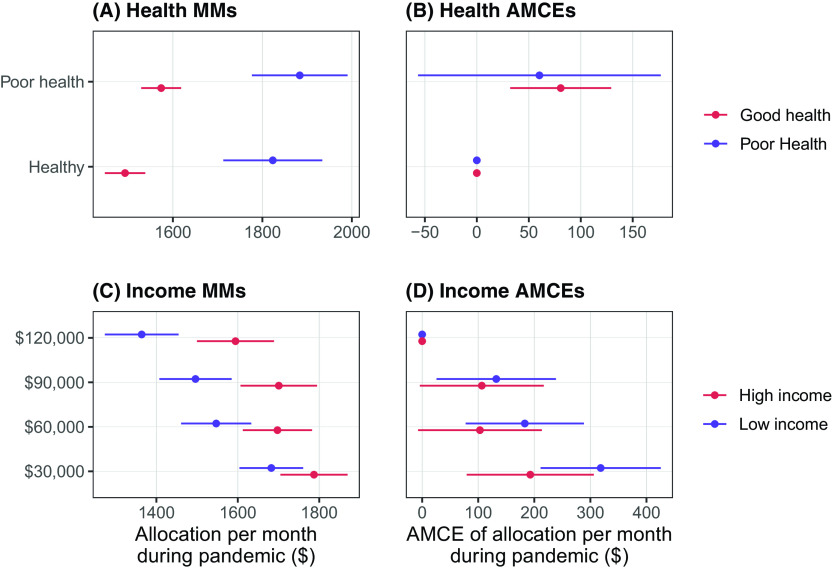
Sub-group marginal means for health status (A) and income (C). Sub-group AMCEs for health status (B) and income (D). *NOTE*: 95% Confidence Intervals are Shown.

Figure 3 shows the same estimates, but for our tests related to health status and pre-pandemic income. The effect of preexisting health conditions is similar for those in good health and those who have experienced recent illness (Figure 3A). This latter group, however, is more generous on average (Figure 3B). There is some indication that respondent income influences distributional preferences. Figure 3C shows that low-income respondents (annual family income $0–60,000) appear to be more sensitive in their deservingness evaluations to pre-pandemic income, comparatively awarding more aid to low-income profiles even while they are less generous than high-income respondents (annual family income greater than $90,000) across all categories. However, the subgroup AMCEs are not significantly different (Figure 3D). Finally, we ran similarity tests for gender and marital status and find further null results, as shown in the Supplementary Materials.

## Study 1 Discussion

Study 1 provides evidence indicating strong support for government cash transfers to Canadian residents among the Canadian population. Critically, we found that ethnic and immigration-based considerations do not strongly drive deservingness (although there is a penalty for noncitizens). Instead, where allocations differed, they did so in ways consistent with the deservingness literature: features like pre-crisis income, children in the household and their ages, income loss, and risk for health complications were important. These effects were generally smaller than those found in the extant literature, however. Finally, Canadians do not appear to be allocating support based on similarity concerns. Those who have been more affected by the crisis (whether through job loss, low pre-crisis income, or illness) do generally support larger transfers, but this largess extends to all potential recipients and not only to those like them.

The COVID-19 pandemic is causing near-universal economic and social dislocation. During this period, support for government aid was high and not subject to previously observed prejudicial or similarity-based considerations, while only being weakly related to deservingness criteria. How to explain these findings? We consider the possibility that, during times of crisis, people may be primed with concerns about national well-being so that redistributive preferences evidence a powerful collective sentiment. If so, the findings from Study 1 provide nuance to existing literature on how attitudes toward redistribution are informed by prejudice or ethnocentrism, as well as social affinity or material self-interest.

Additional evidence is required to support such a conclusion, however. Are disaster-related cash transfers truly different from other government programs? Are deservingness and similarity considerations weaker for such crisis spending? To do so, we registered a second study featuring a modified conjoint design. We hypothesize:

Support for COVID-19 cash transfers will be more universal and less subject to prejudicial, deservingness, and similarity considerations.

## Study 2: Deservingness and direct cash transfers

Study 2 differs from Study 1 in four important ways. First, respondents were randomly assigned to either a question set focused on the COVID-19 cash transfer or a COVID-unrelated cash transfer (here a Goods and Services Tax rebate, hereafter GST). The precise wordings of each condition are found in Table 1.^5^ Second, profile names were sampled without replacement in order to prevent respondents from seeing the same profile name twice. Third, due to concerns about framing effects, we replaced the sliding scale used by respondents in the pilot with a blank text box that respondents filled in with a dollar amount of their choosing.

**Table 1:** Study 1 and 2 Designs (conjoint features bolded)

Description	Outcome
Study 1	
In the following section, we would like you to read about people living in Canada during the current COVID-19 pandemic. Please read about each person’s situation, then tell us how much government support, if any, you think they should receive per month during the pandemic. **Name** was citizenship. They are currently living in a large Canadian city. They are **marital status** and have **children**. **Name** is **employment status**. They are **health status**. Prior to the COVID-19 pandemic, **Name**’s income was **income**.	How much money should name receive per month from the government during the pandemic? ($0–$4000 selection bar, with default position at $0)
**Study 2** - random assignment to (A) or (B)	
**(A):** The COVID-19 pandemic increased Canadian unemployment to a record 13.7%, with millions of Canadians out of work. Over 120,000 Canadians have contracted the disease and approximately 9,000 Canadians have died. In the following section, we would like you to read about people living in Canada during the current COVID-19 pandemic. Please read about each person’s situation and think about the financial support they may need from the government. After each pair of profiles, we will ask you some questions. **Name** is **citizenship** and is of **ethnicity** descent. They currently live in a large Canadian city. They are **marital status** and have **children**. **Name** is **employment status**. They are **health status**. Their income in 2019 was **income**.	**(1)** In general, how similar is **Name** to you? (0-10 scale, with 0 labelled very different, and 10 labelled very similar) **(2)** How deserving is **Name** of government financial support (in the form of a government GST rebate OR during the pandemic)? (0-10 scale, with 0 labelled very undeserving, and 10 labelled very deserving) **(3)** How much money, if any, should **Name** receive per month from (the government GST rebate program OR the government during the pandemic)? (Empty box with numeric whole number validation) Note that respondents received the question on allocation (3) either before or after the similarity and deservingness questions before OR after the allocation question (randomly assigned).
**(B):** The GST rebate is a monthly government tax rebate provided to Canadians. It is not related to special pandemic support, existed before the current COVID-19 pandemic, and there are no plans to cancel it after the pandemic is over. In the following section, we would like you to read about people living in Canada. Please read about each person’s situation and think about the financial support they may need from the government. After each pair of profiles, we will ask you some questions. **Name** is **citizenship** and is of **ethnicity** descent. They currently live in a large Canadian city. They are **marital status** and have **children**. **Name** is **employment status**. They are **health status**. Their income in 2019 was **income**.	

Fourth, two additional outcomes were measured: subjective similarity and a direct evaluation of deservingness (taken from Kootstra 2016). Study 1 suggested a period of unusually high social solidarity where similarity does not drive the allocation decision: we found that objective measures of similarity were nonfactors in allocation decisions. However, in the analysis of Study 1, the similarity was inferred based on objective criteria, whereas respondents may interpret their similarity to the profiles in a different manner than the original model supposed. For example, racial considerations might be the most important for a given respondent while for others the presence or absence of children may be primary.

A second outcome measure was captured that directly evaluates the respondent’s perceptions of deservingness. The literature suggests that citizenship and ethnicity are likely to guide perceptions of deservingness, and both the literature and our pilot suggest that practical considerations such as employment status, number of children, pre-crisis income, and health status are likely to structure decisions about deservingness. The addition of these outcome measures also necessitated a shift from a paired to a single profile design given increased cognitive demands of the task. The order of the outcomes was randomized (either the allocation question first or last) to avoid priming effects.

The second study was fielded from November 22 to December 13, 2020 using the same survey provider as Study 1 with a sample of 2,501 respondents.^6^ Each respondent was randomly assigned either a COVID-19 or a GST condition and received four profiles. We screened out those specified in the registration: straight liners, those who completed the survey less than a third of median completion time, and those who allocate unreasonable dollar amounts to the profile (above $9,999 per month with subsequent removal of the top 2% of responses).

### Study 2 Results

**Figure 4:**
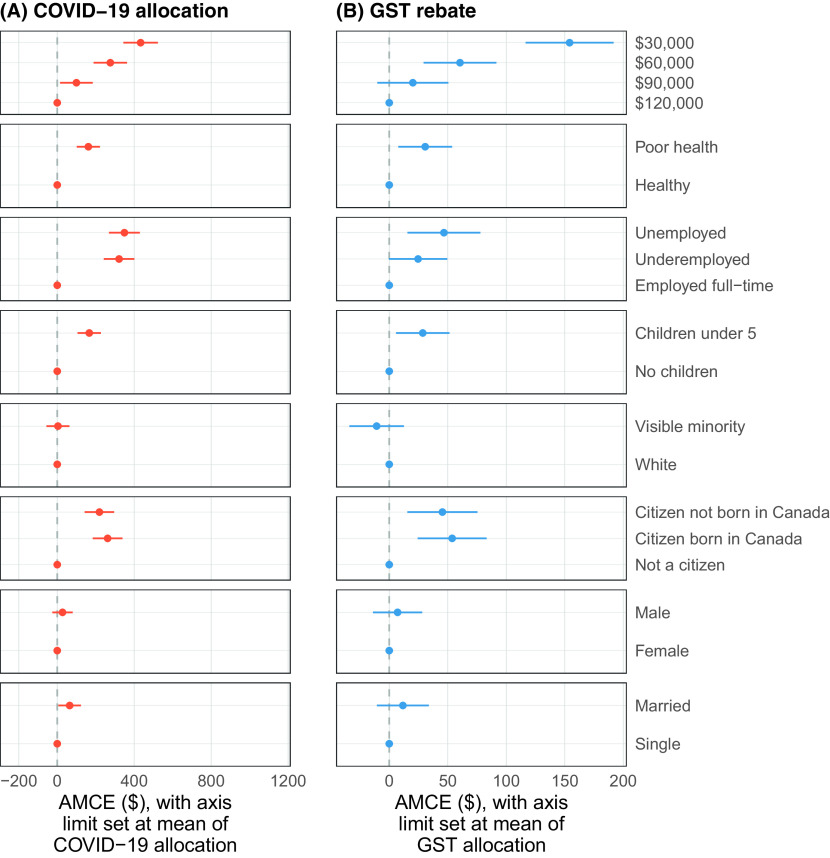
(A) AMCEs under COVID-19 Cash Transfer and (B) GST Rebate Conditions. *NOTE*: 95% Confidence Intervals are Shown.

We first present findings similar to the Study 1. Figure 4 shows the AMCE results for COVID-19 (A) and GST (B). We observe, consistent with Study 1, that common deservingness characteristics (previous income, children, employment, health, and citizenship status) are important considerations for respondents under both the COVID-19 and GST conditions. Also consistent with Study 1, ethnicity, gender, and marital status had no effect on the allocations.

**Figure 5:**
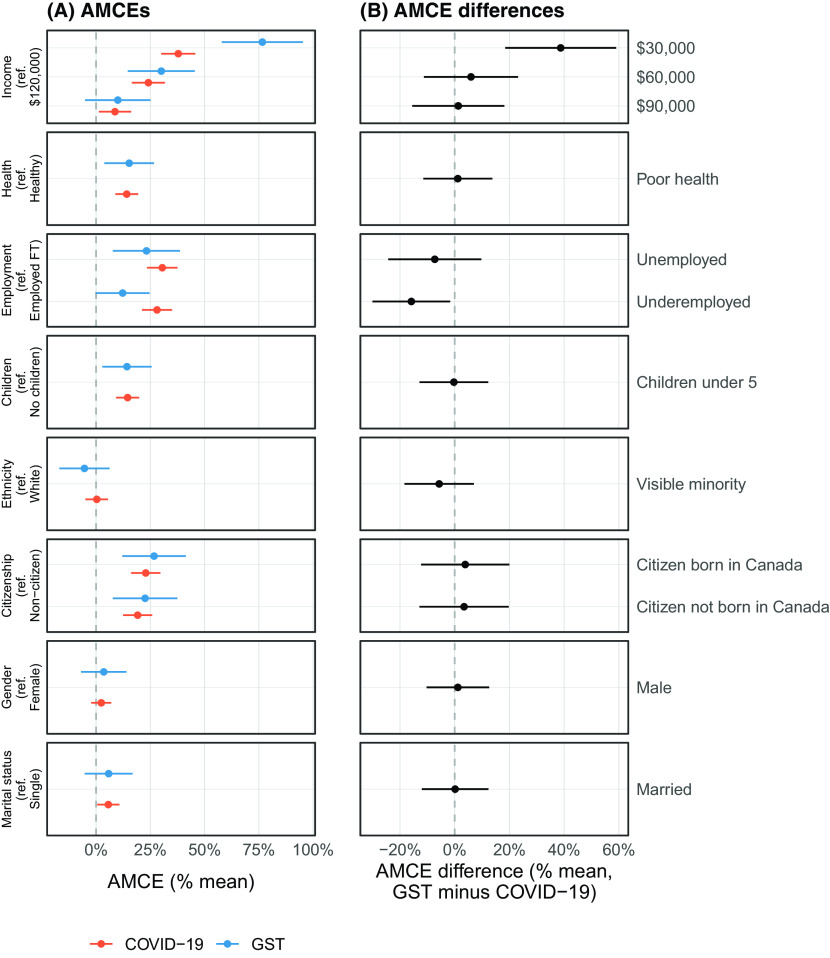
(A) Mean-Normalized AMCEs for COVID-19 and GST Conditions and (B) Mean-Normalized AMCE Differences. *NOTE*: 95% Confidence Intervals are Shown.

We next consider our main hypothesis for Study 2: support for COVID-19-related spending is more universal as compared to a more general government cash transfer program (the GST rebate). Formally, we anticipated that deservingness features in the planned experiment will have stronger causal effects on allocation amount for the GST condition respondents. Given that the mean allocation for COVID-19-related relief was higher than that of GST, we show mean-normalized allocation amounts for comparability between the two conditions in Figure 5. Figure 5(A) shows mean-normalized AMCE values for both the COVID-19 and GST conditions while Figure 5(B) shows mean-normalized AMCE differences between the two experiments. We find that only one feature differentially substantially affects cash allocations between COVID-19 and the GST rebate: the lowest income category. All other features that matter for allocations equally structure both forms of transfers. We find partial evidence that deservingness considerations structure respondents allocations more for non-crisis-related spending.

We report an *F*-test comparing model with and without interaction terms between the randomly assigned condition and the profile features. We exclude those variables where no effects are expected (gender and marital status). We find an *F*-statistic of 2.7 (*p* = 0.002), which allows a confident rejection of the null that there are no differences between expected allocation amounts under the COVID-19 and GST conditions (contingent upon the conjoint feature set). Again, it is the income category that drives these results and no other feature.

**Figure 6:**
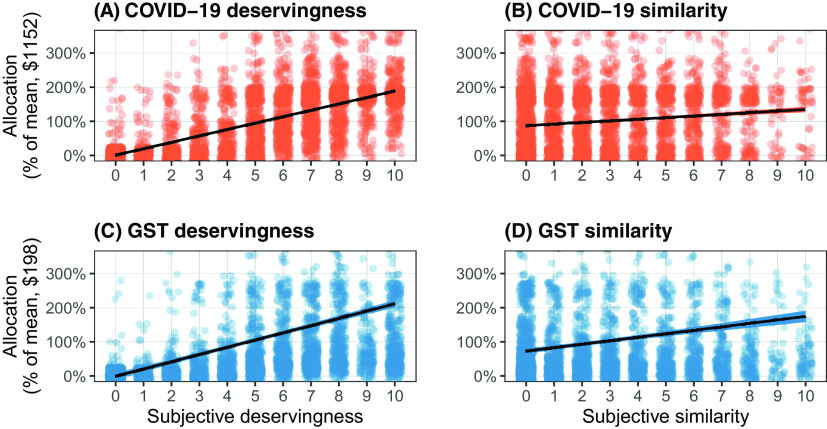
Associations between Deservingness (A and C), Similarity (B and D) and Allocation Amount.

Next, we examine the relationship between subjective evaluations of similarity and deservingness and the dollar amount allocated to a given profile in both the COVID-19 and GST conditions. Note that similarity and difference are not experimentally assigned, but instead measured within the context of the experiment. Figure 6 shows the correlations between similarity/deservingness and the allocation for both the COVID-19 allocation and the GST rebate. Figure 6 (A) and (C) on the left show allocation as a function of deservingness for COVID-19 and the GST rebate, while Figure 6(B) and (D) on the right show the same for subjective similarity. We anticipated both deservingness and similarity effects, with perceived deservingness to be the primary driver of allocation.

We find that deservingness is a far stronger predictor of allocation amount, while subjective similarity is only somewhat positively associated with increased allocations (a relationship that vanishes when controlling for deservingness). Moreover, the relationship between deservingness and the GST allocation is somewhat stronger than that between deservingness and the COVID-19 allocation.

Table 2 presents the formal findings using three model specifications: regressing allocation directly on deservingness and similarity, including individual respondent fixed effects (to account for heterogeneity in government support preferences). Model 1 shows how deservingness and similarity considerations affect allocation for COVID-19 direct cash transfers. Model 2 shows the same for the GST rebate. Values in Models 1 and 2 are not normalized or directly comparable, however, Model 3 shows results for both conditions combined under dependent variable mean normalization. We find, as expected, a positive interaction for GST × Deservingness, which indicates that deservingness considerations weigh more heavily in allocation decisions for the GST condition. Conversely, deservingness is less likely to structure cash allocations in response to COVID-19, which we attribute to support for a more universal approach during a crisis situation.

**Table 2.** Subjective Evaluations of Deservingness and Similarity

	COVID-19	GST Rebate	Mean-Normalized
Constant	594.80 (249.56)	− 122.40 (109.86)	− 0.17 (0.42)
GST			− 0.44 (0.59)
Deservingness (0–10)	202.60 (3.94)*	41.36 (1.55)*	0.18 (0.01)*
Similarity (0–10)	− 2.98 (4.91)	0.97 (1.95)	− 0.00 (0.01)
GST × Deservingness (0–10)			0.03 (0.01)*
GST × Similarity (0–10)			0.01 (0.01)
*R* ^2^	0.83	0.77	0.78
Adj. *R* ^2^	0.76	0.69	0.70
Num. obs.	4243	4308	8551

*Notes*: *p < 0.01. Linear regression for subjective evaluations of deservingness and similarity with individual respondent controls and clustered standard errors in parentheses. Dependent variable: allocation of cash transfer to hypothetical individuals.

These three sets of analyses collectively evaluate the role that subjective deservingness and similarity play in allocation decisions and better explore how those evaluations guide decisions about direct cash transfer assistance. Figure 4 provides evidence that it is practical concerns such as employment status and number of children that drive deservingness considerations both during the pandemic and in general. Figure 5 and the associated *F*-test provide evidence that deservingness considerations are less relevant for crisis-related cash transfers (but only due to income-related factors). Finally, Figure 6 and Table 2 provide evidence that the allocation is powerfully determined by deservingness but not similarity evaluations, but that these deservingness effects are weaker for those asked about a COVID-19 transfer.

## Discussion

Governments around the world have provided unprecedented and generally universal support to individuals adversely affected by the crisis. In Canada, these dramatic and broad cash transfers are widely supported by the mass public at levels that compare to those which the government distributed during the first and second waves of the pandemic.

While previous literature has shown that ethnic and immigration-based considerations are important determinants of allocation decisions, we do not find such effects during the pandemic (albeit with small penalties for noncitizens). Rather, where allocations differ they do so in relatively uncontroversial ways, such as giving more to those who are ill, who have children, or have lower incomes. Moreover, while objective measures of similarity between respondents and hypothetical recipients have been previously shown to impact allocations, again we find no effects during the pandemic. In two studies, we have shown that neither objective nor subjective similarity matter for either COVID-19-related allocations or more general allocations during the time of the pandemic. At least two possibilities explain the discrepancy with previous literature: (1) the same universal approach to financial support applies to all government programs in the midst of a pandemic or (2) the importance of similarity may have faded since previous studies and now is not a relevant consideration guiding generosity toward government assistance recipients. Future research taking place in a post-pandemic world can evaluate these two explanations.

Deservingness, measured either with objective or subjective criteria is found to be important for allocation decisions, however. Health status, children, citizenship, employment status, and income are all relevant considerations for government spending. In Study 1, however, we find somewhat smaller effects for objective attributes of deservingness than we anticipated. Study 2 allowed us to compare the sizes of these effects for pandemic-related spending and for a preexisting government cash transfer under the expectation that they mattered more for the latter. We find this to be the case, but only for income. The tendency to allocate more aid to lower income profiles relative to the wealthy was considerably stronger among those in the GST rebate condition. We further find that subjective evaluations of deservingness matter more for GST rebate allocations. As expected, the tendency toward universalism is stronger in attitudes toward pandemic-related government aid. Note that this universalism produces a redistributive schema that is net less progressive (in that it applies equally across income categories).^7^

The implication of this finding is considerable. In times of crisis, citizens adopt more universalist attitudes toward redistribution. They are willing to grant a large amount of government aid to individuals across the entire income distribution and are less willing to claw back aid for wealthier individuals. Governments, then, have sizeable scope to adopt large-scale, universal cash transfers with little worry of public backlash in these circumstances.

There are several important limitations to the above analysis that could serve as a launching point for future research. The conclusions drawn from the observed differences between the COVID-19 and GST allocations are subject to two main caveats that require further testing. First, our experiments were conducted at a time when COVID-19 was affecting every aspect of our respondents’ lives. Respondents were likely pre-treated with universalist/collectivist themes from political discourse, which carried into their allocation decisions for both the COVID-19 cash transfers as well as the GST rebate. Further, some respondents may well view the GST rebate as a tool to provide pandemic relief (indeed it has been used to supplement other COVID-19 relief funding in Canada). We took pains to minimize this problem by putting the experiment at the beginning of the survey, providing information in the COVID-19 aid condition emphasizing the costs of the pandemic, and emphasizing that we were asking about non-pandemic-related government aid in the GST rebate condition. However, we still view the differences we observe as conservative in nature. COVID-19 may have played an even bigger role in changing attitudes toward government aid.

Second, the mean COVID-19 relief allocation was significantly larger than that of the GST rebate and these anchoring effects may have partially driven the AMCE difference observed in Figure 5. In other words, redistributive preferences may differ based on the absolute dollar size of the program. We think this is highly unlikely for two reasons: (1) mean-normalized AMCEs between the two conditions were generally similar for non-income deservingness features such as children, employment, health, and citizenship status; and (2) subjective evaluations of deservingness offer more explanatory power for the allocation under the GST condition, which provides strong evidence for an underlying dynamic as opposed to measurement error induced by anchoring effects. Regardless, subsequent research could investigate the extent to which the size of a redistributive program is an important consideration for allocation decisions based on deservingness.

In addition to these two caveats, we are unable to unpack the causal mechanism through which COVID-19 affects redistributive preferences. Is the COVID-19 pandemic priming people with universalist/collectivist themes because of a sense of loss that they then lean on when making allocation decisions? Or are there more practical considerations, like using government aid to keep as many people home as possible to minimize the spread of the virus? Future experiments could randomly assign information with instrumentalist or collectivist themes to see how these affect allocation decisions.

During the first two waves of the COVID-19 pandemic in Canada, support for government aid was high, universal, and unencumbered by typical considerations such as deservingness or similarity. These findings provide nuance to existing literature on how attitudes toward redistribution are informed by prejudice or ethnocentrism, as well as social affinity or material self-interest. During times of crisis, people may be primed with concerns about national well-being, with redistributive preferences driven by a powerful collective sentiment that citizens are “all in this together”.

## Data Availability

The data, code, and any additional materials required to replicate all analyses in this article are available at the Journal of Experimental Political Science Dataverse within the Harvard Dataverse Network, at: https://doi.org/10.7910/DVN/LPHMB7.

## Footnotes

1 For example, the Canada Emergency Response Benefit (CERB) was specifically designed to be quickly rolled out (Government of Canada Hansard March 24, 2020).

2 In Canada, the largest of these programs is the Canadian Emergency Response Benefit, which is a taxable benefit of $500 per week made available to Canadians who met eligibility requirements (workers who stopped working or whose work hours were reduced due to COVID-19). This is an amount approaching the maximum unemployment insurance benefit in Canada ($573 per week). These benefits were made available for a period of 16 weeks (later extended to 24 weeks). Other cash transfer programs included a one time increase in the Canada Child Benefit for a total of $300 per child, an increase in the Goods and Services Tax (GST) credit, and $1,250 per month available for students who were unable to find summer employment.

3 This design uses a single concrete measure as opposed to a more abstract survey measure (e.g. measuring support for spending with an item similar to: *The government should take measures to reduce differences in income levels*). To test the full range of deservingness, similarity, and prejudicial factors, a design with a concrete measure is most appropriate.

4 See the preregistration of this design and associated hypotheses at https://osf.io/2rqwm.

5 The features and levels of both Study 1 and Study 2 are found in the Supplementary Materials.

6 A full registered report is available at https://osf.io/x9642. In addition, a third study was run after submission but prior to receipt of peer review suggestions. Space was available in a survey being fielded and we used the design originally submitted for peer review. This survey was preregistered on July 13 and was fielded from July 22 to July 29, 2020, with 2,315 respondents meeting the preregistered attention checks and validation on the allocation amounts. The results of Study 2 replicate in this third study despite design changes and different sampling periods, providing additional evidence for these findings. See the Supplementary Materials for a discussion of the design differences and the full results from this third study.

7 We conduct two non-preregistered analyses in the Supplementary Materials to provide additional evidence for this more universal, but ultimately regressive disaster relief spending. First, we examine the AMCEs and AMCE differences for deservingness. We find that perceived deservingness is very strongly influenced by income of the recipient, with the tendency stronger for the GST condition. Conversely, COVID-19 relief deservingness is relatively less influenced by pre-crisis income. Second, we find evidence of a triple interaction where GST condition × Deservingness × low hypothetical income drives differences in allocation amounts.

## Supplementary material

For supplementary material accompanying this paper visit http://dx.doi.org/10.1017/XPS.2021.10. click here to view supplementary material
