# Supplementary material for: All in This Together? A Preregistered Report on Deservingness of Government Aid During the COVID-19 Pandemic
Source: Journal of Experimental Political Science. 2021 Mar 31:1–18. doi: 10.1017/XPS.2021.10 (PMC8207562; doi:10.1017/XPS.2021.10)
Supplement: Supplementary file 1 [file xpssup.zip › S2052263021000105sup001.pdf]

# Supplementary materials

## All in this together? A preregistered report on deservingness of government aid during the COVID-19 pandemic

Aengus Bridgman, Eric Merkley, Peter John Loewen, Taylor Owen, and Derek Ruths

**A Study 1 objective similarity measures** i

**B General support for redistribution** iii

**C Pre-peer reviewed experiment** vi

**D Study factorial levels** xi

**E Additional exploratory analysis of deservingness** xiii

# A Study 1 objective similarity measures

Given multiple levels of the vignette features and respondent characteristics, the following design choices were made to identify social affinity or material self-interest considerations for Study 1.

Table A1: Similarity considerations

| Variable | Question wording and construction | Category | % |
|---|---|---|---|
| Employment at risk | Do you have a job at risk because of the coronavirus? | Yes | 19.8 |
| | | Maybe | 16.8 |
| | | No | 56.9 |
| | | Laid off | 6.5 |
| Employment at risk (dichotomized) | 1 = Yes OR laid off | | 27.0 |
| Children | How many children do you have? | 0 | 46.6 |
| | | 1 | 19.9 |
| | | 2 | 22.2 |
| | | 3 | 7.3 |
| | | 4 | 2.4 |
| | | More than 4 | 1.7 |
| Children (dichotomized) | 1 = 1 OR 2 OR 3 OR 4 OR more than 4 | | 54.1 |
| Recent experience with illness (personally) | 1=sore throat OR difficulty breathing/shortness of breath OR fever OR a severe dry cough OR loss of sense of sell OR a fever with hallucinations | | 14.1 |
| Recent experience with illness (household) | 1=sore throat OR difficulty breathing/shortness of breath OR fever OR a severe dry cough OR loss of sense of sell OR a fever with hallucinations | | 9.9 |
| Recent experience with illness (combined) | 1=personal OR household experience | | 17.2 |
| Income | Total household income | $0-60,000 | 40.3 |
| | | $60,001-90,000 | 20.3 |
| | | $90,0001+ | 39.4 |
| Marital status | 1 = married | | 46.3 |
| Gender | 1 = female | | 51.4 |

No sub-group AMCE effects were identified for gender or marital status, as shown in Figure A1.

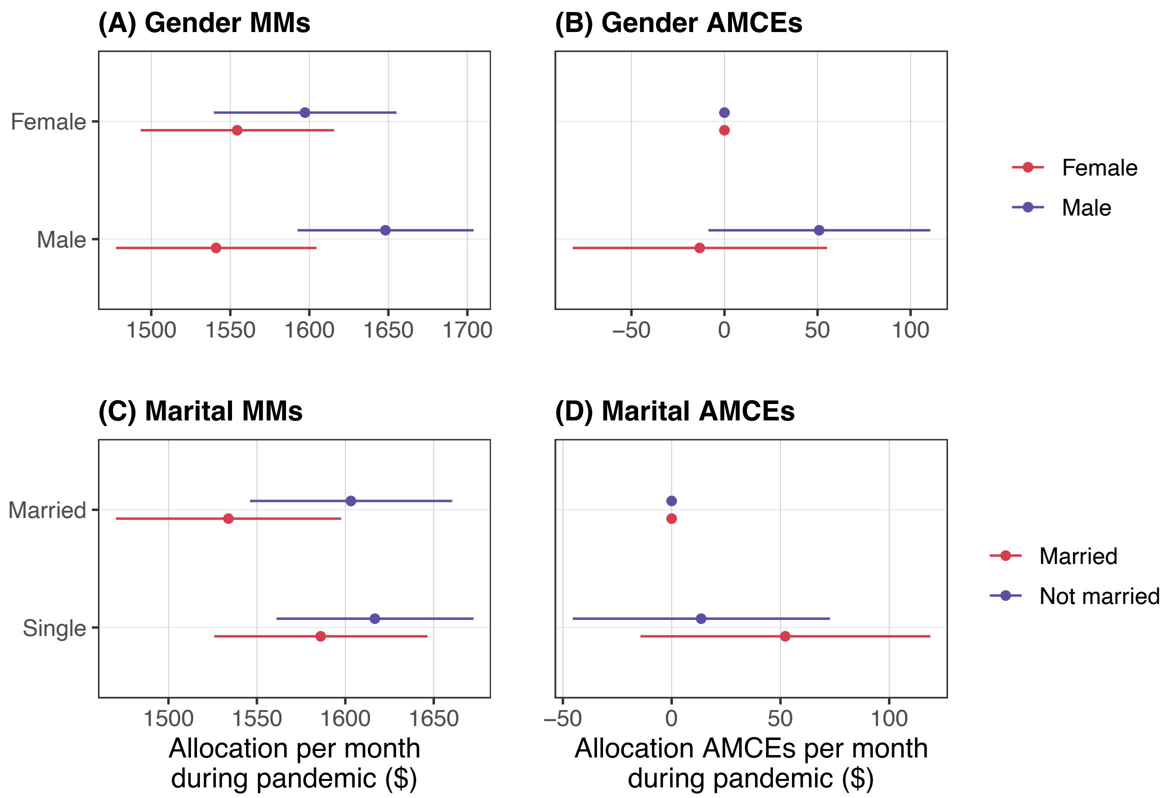


Figure A1: Sub-group marginal means for gender (panel A) and martial status (panel C). Sub-group AMCEs for gender (panel B), marital status (panel D). 95% confidence intervals shown.

## B General support for redistribution

We evaluated the relationship between overall support for government redistribution related to COVID-19 using responses to a set of questions asking about support for Government measures taken during the pandemic. The exact item used is: *Governments have taken a variety of actions in response to COVID-19. To what extent do you support or oppose the following?*, with four COVID-19 specific transfers measured. We create an index by averaging across the four measures and then show the associations in Figure A2: 1) deservingness in panel A; 2) similarity in panel B; 3) COVID-19 allocations in panel C; and 4) GST in panel D.

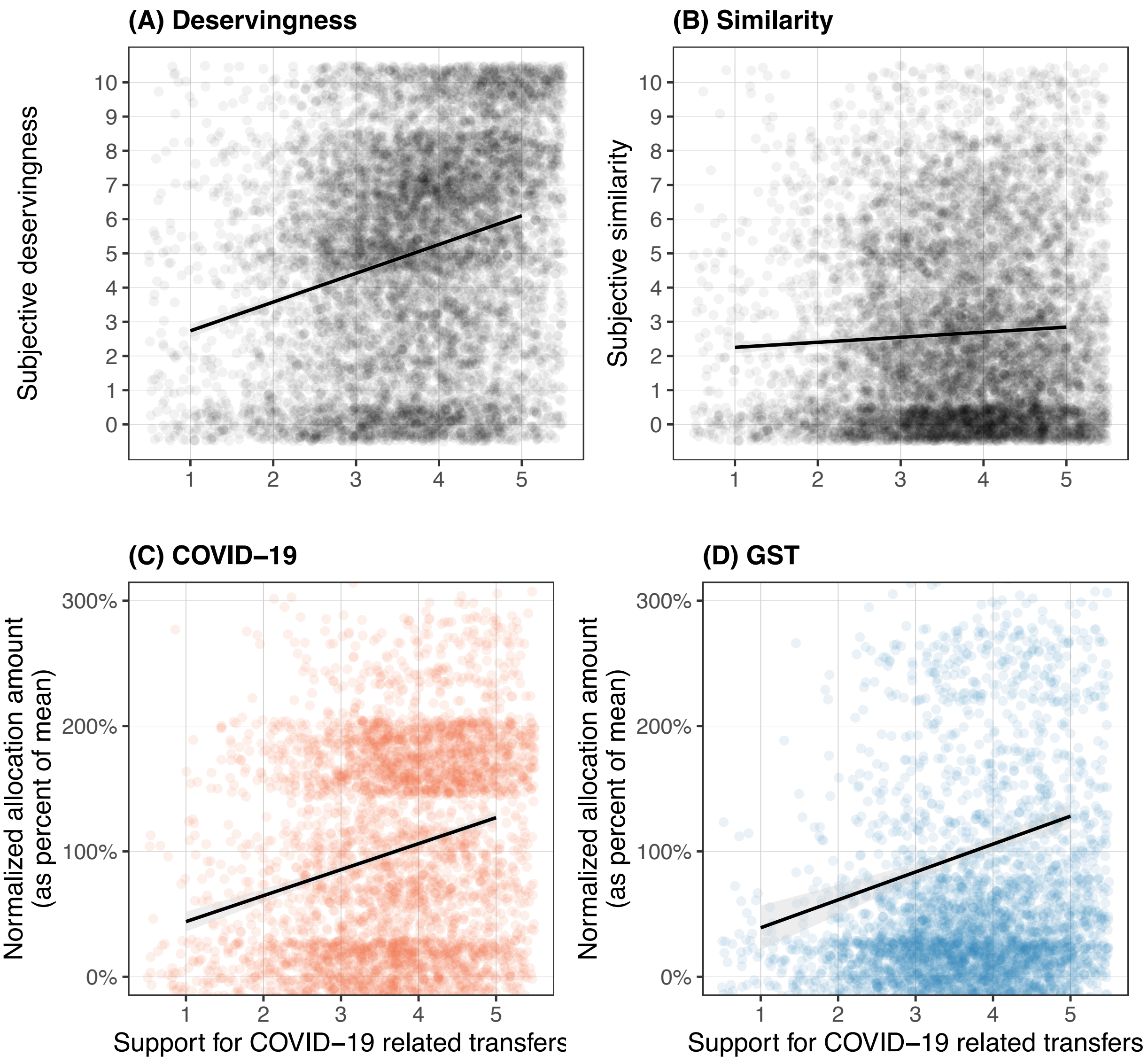


Figure A2: Associations between outcome measures and overall support for COVID-19 related government spending.

We find strong positive associations between overall support and determinations of

Table A2: Correlations between mean-normalized allocations and general support for government redistribution programs

| | Model 1 | Model 2 | Model 3 | Model 4 |
|---|---|---|---|---|
| Constant | 0.36 (0.06)* | 0.54 (0.06)* | 0.63 (0.05)* | 0.48 (0.07)* |
| Canadian Emergency Response Benefit | 0.16 (0.01)* | | | |
| Canadian Emergency Wage Subsidy | | 0.12 (0.02)* | | |
| General support for universal cash transfers | | | 0.10 (0.01)* | |
| General support for Employment Insurance enhancements | | | | 0.13 (0.02)* |
| $R^2$ | 0.01 | 0.01 | 0.01 | 0.01 |
| Adj. $R^2$ | 0.01 | 0.01 | 0.01 | 0.01 |
| Num. obs. | 8338 | 8199 | 8071 | 8293 |

$^*p < 0.01$. Dependent variable: mean-normalized allocation of cash transfer to hypothetical individuals, either under the COVID-19 or GST conditions

deservingness and the two allocation amounts. Supporting government assistance more broadly is associated with individuals evaluating potential government aid recipients as more deserving. This suggests that perceived deservingness is not simply a function of the recipients characteristics but also overall attitudes towards government spending.

We further show correlations between support for particular government spending programs and normalized allocation in Table A2 and general attitudes towards redistribution in Table A3. All relationships are as expected.

Table A3: Correlations between mean-normalized allocations and attitudes about redistribution

| | Model 1 | Model 2 | Model 3 |
|---|---|---|---|
| Constant | 1.15 (0.05)* | 0.76 (0.05)* | 0.72 (0.06)* |
| People who don't get ahead should blame themselves, not the system | −0.05 (0.01)* | | |
| Government should take measures to reduce differences in income levels | | 0.07 (0.01)* | |
| The government should see to it that everyone has a decent standard of living | | | 0.07 (0.02)* |
| $R^2$ | 0.00 | 0.00 | 0.00 |
| Adj. $R^2$ | 0.00 | 0.00 | 0.00 |
| Num. obs. | 8365 | 8327 | 8443 |

*$p < 0.01$. Dependent variable: mean-normalized allocation of cash transfer to hypothetical individuals, either under the COVID-19 or GST conditions.

## C Pre-peer reviewed experiment

A version of the Study 2 experiment was run prior to the suggestions of peer reviewers of the registered report. Space was available in a survey being fielded and we used the design originally submitted for peer review. This survey was preregistered on July 13 (https://osf.io/x9642) and was fielded from July 22 to July 29, 2020, with 2315 respondents meeting the pre-registered attention checks and validation on the cash allocations. The results are extremely similar to those of the revised (as per reviewer recommendations) design. This is despite these four differences between the two experiments:

1. A stronger prompt on the GST condition. The peer reviewed experiment used: "The GST rebate is a monthly government tax rebate provided to Canadians. It is not related to special pandemic support, existed before the current COVID-19 pandemic, and there are no plans to cancel it after the pandemic is over", while the original design simply stated: "The GST rebate is a monthly government tax rebate provided to Canadians".

2. Profiles having a more explicit prompt to test whether or not racial considerations structured transfer allocations. The pre-peer reviewed experiment read: "**Name** is **citizenship**", while the final version read: "**Name** is **citizenship** and is of **Chinese/Indian/Indigenous/European** descent."

3. Non-replacement sampling of hypothetical recipient Name. We removed the possibility of a respondent seeing the same name when considering the hypothetical recipients. In the pilot and pre-peer reviewed study the names were sampled with replacement. We computed the likelihood of duplication occurring and, somewhat counterintuitively, approximately 60% of respondents would see at least one duplicate name if 4 samples are pulled from only 8 possibilities. In the final version of the study participants saw a name a maximum of one time.

4. Randomization of outcomes. The pre-peer reviewed experiment measured similarity, then deservingness, then the cash allocation. The final version randomized the order, showing either the cash allocation or the similarity and deservingness questions first.

We have run the same analysis on the data from the pre-peer reviewed experiment. Results are shown and briefly described below. Figure A3 show AMCE results for the COVID-19 and GST conditions. We observe, consistent with Study 1 and 2, that common deservingness characteristics (previous income, children, employment, health, and citizenship status) are important consideration for respondents under both the COVID-19 and GST conditions. Also consistent, ethnicity, gender, and marital status had little effect on the allocations. Under the COVID-19 condition, however, respondents allocated more to hypothetical male recipients.

Next we show mean-normalized AMCE differences. Similar to Study 2, we find that the lowest income category deferentially affects cash allocations between COVID-19 and the GST rebate. Unlike Study 2, we also find small effects for poor health and the presence of children under the age of 5.

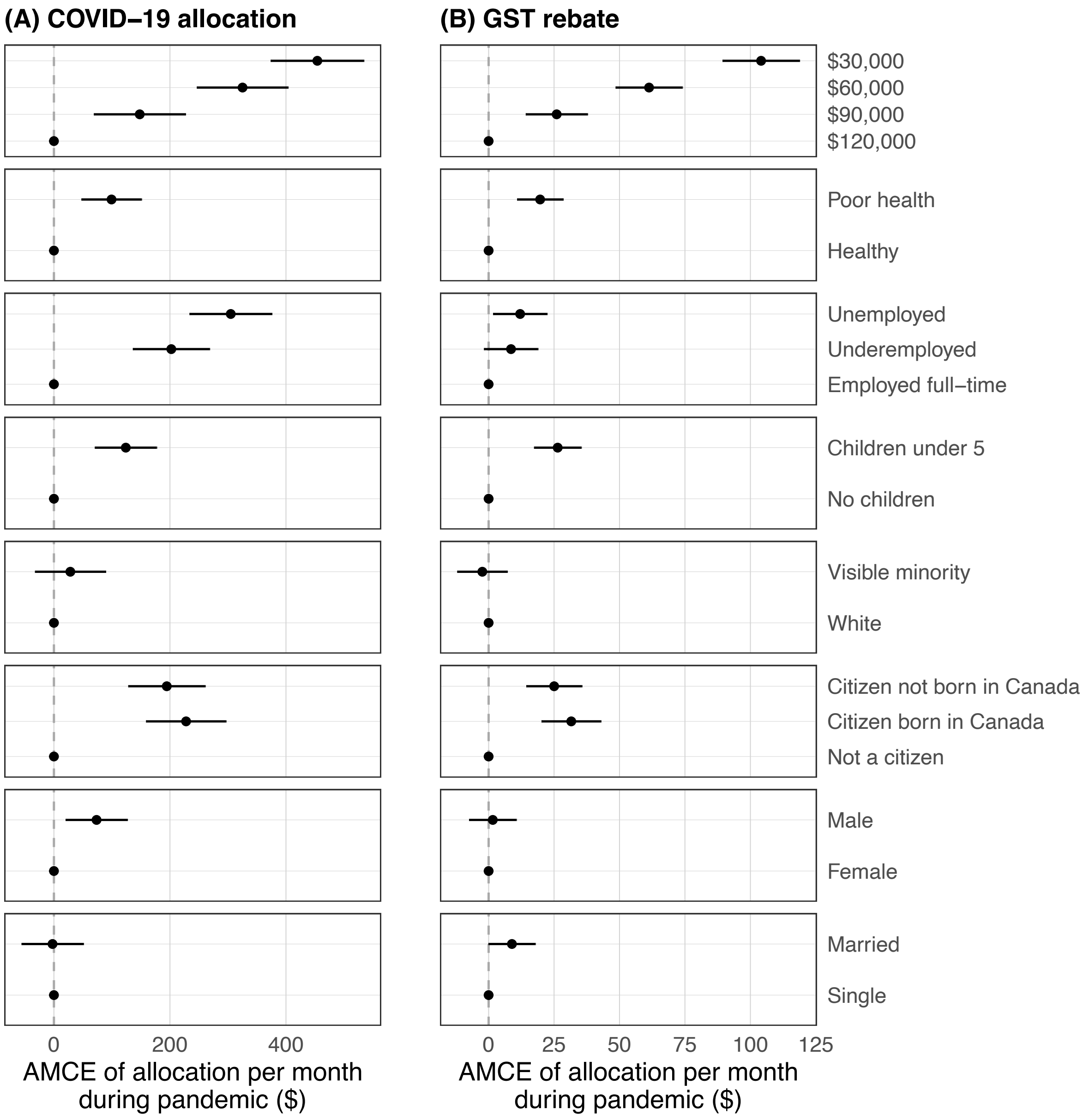


Figure A3: AMCE results of allocation under COVID-19 allocation (panel A) and GST rebate (panel B) conditions. 95% confidence intervals shown.

We report the same F-test comparing models with and without interaction terms between the randomly assigned condition and the profile features. For this study, we find an F-statistic of 3.3 ($p = 0.0004$), which allows a (stronger than Study 2) confident rejection of the null that there are no differences between expected allocation amounts under the COVID-19 and GST conditions (contingent upon the conjoint feature set). Again, it is the income category that drives these results and no other feature.

Next, we find that the relationship between subjective evaluations of similarity and deservingness and the dollar amount allocated to a given profile in both the COVID-19

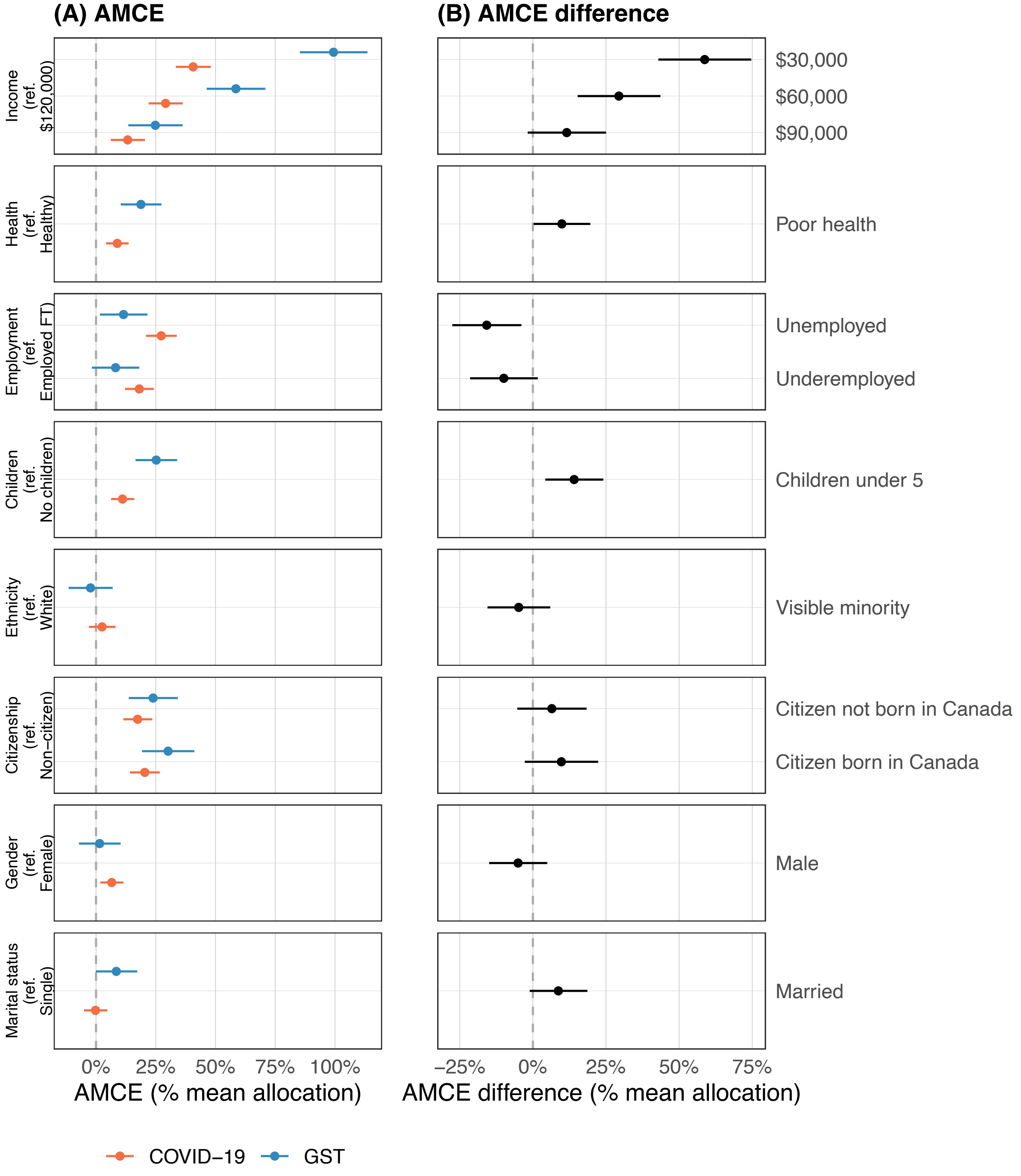


Figure A4: Results for mean-normalized AMCEs for COVID-19 and GST conditions (panel A), and mean-normalized AMCE differences (panel B). 95% confidence intervals shown.

and GST conditions are again very similar. Figure A5 shows the correlations between similarity/deservingness and the allocation for both the COVID-19 allocation and the GST rebate. We find that deservingness is the far stronger predictor of allocation, while sub-

jective similarity is only somewhat positively associated with increased allocations (a relationship that vanishes when controlling for deservingness). The relationship between deservingness and the GST allocation is similarly stronger that that between deservingness and the COVID-19 allocation.

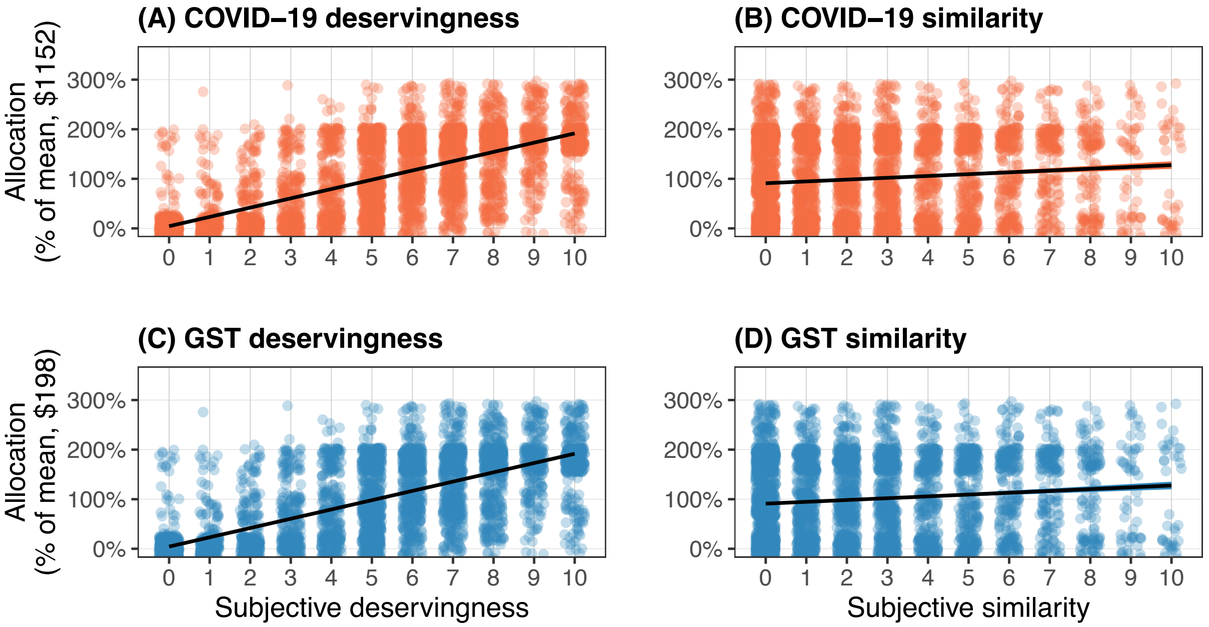


Figure A5: Associations between deservingness (panels A and C)/ similarity (panels B and D) and allocation amount.

Table 2 presents the regression findings. We find similar coefficients for deservingness on both COVID-19 (202.60 as compared to 209.28) and the GST conditions (33.64 and 41.36). Similarity is not significant in the final study, but shows a very small negative effect in the pre-peer reviewed study (-3.97). The mean-normalized coefficients are similar for deservingness (0.18 and 0.18), and deservingness is more powerful associated with allocation under the GST condition (0.03 vs 0.04).

The near exact replication despite design changes and different sampling periods indicates additional robustness to these findings.

Table A4: Subjective evaluations of deservingness and similarity

| | COVID-19 | GST rebate | Mean-normalized |
|---|---|---|---|
| Constant | 15.82 (224.08) | −3.48 (43.45) | 0.01 (0.32) |
| GST | | | −0.01 (0.45) |
| Deservingness (0-10) | 202.16 (3.60)* | 25.49 (0.65)* | 0.18 (0.01)* |
| Similarity (0-10) | −12.06 (4.35)* | −1.88 (0.88) | −0.01 (0.01) |
| GST x Deservingness (0-10) | | | 0.06 (0.01)* |
| GST x Similarity (0-10) | | | −0.01 (0.01) |
| $R^2$ | 0.83 | 0.77 | 0.79 |
| Adj. $R^2$ | 0.77 | 0.69 | 0.71 |
| Num. obs. | 4420 | 4181 | 8601 |

*$p < 0.01$. Linear regression for subjective evaluations of deservingness and similarity, with individual respondent controls and clustered standard errors in parentheses. Dependent variable: allocation of cash transfer to hypothetical individuals, either under the COVID-19 or GST conditions.

## D Study factorial levels

Table A5: Factorial levels for Study 1

| Feature | Levels |
|---|---|
| Name | • Matthew/Laurie Smith (White English) OR Thomas/Emma Gagnon (White French)<br>• Julia/John Wong (East Asian)<br>• Amir/Anya Sandhu (South Asian)<br>• Robert/Linda Blackhawk (Indigenous) |
| Citizenship | • born in Canada and is a Canadian citizen<br>• not born in Canada, but is a Canadian citizen<br>• not born in Canada and is not a Canadian citizen |
| Martial Status | • single<br>• married |
| Children | • no children<br>• 2 children under the age of 5<br>• 2 children between the ages of 5 and 12<br>• 2 children over the age of 12 |
| Employment Status | • employed full-time and has not any loss of wages.<br>• is employed full-time but their wages and hours have been reduced.<br>• is unemployed due to the pandemic<br>• is unemployed for reasons unrelated to the pandemic. |
| Health Status | • They are in good health<br>• They have a pre-existing condition which makes them more susceptible to complications arising from COVID-19. |
| Income | • $30,000<br>• $60,000<br>• $90,000<br>• $120,000 |

Table A6: Factorial levels for Study 2

| **Feature** | **Levels** |
|---|---|
| Name | • Matthew/Laurie Smith (White English) OR Thomas/Emma Gagnon (White French)<br>• Julia/John Wong (East Asian)<br>• Amir/Anya Sandhu (South Asian)<br>• Robert/Linda Blackhawk (Indigenous) |
| Citizenship | • born in Canada<br>• born outside of Canada, but is a Canadian citizen<br>• born outside of Canada and is not a Canadian citizen |
| Martial Status | • single<br>• married |
| Children | • no children<br>• children under the age of 5 |
| Employment Status | • is currently employed full-time<br>• is currently underemployed (is working part-time but cannot find full-time work)<br>• is currently unemployed |
| Health Status | • in good health<br>• in poor health |
| Income | • $30,000<br>• $60,000<br>• $90,000<br>• $120,000 |

# E Additional exploratory analysis of deservingness

We conducted two non-registered analyses utilizing the subjective deservingness measure to better understand the relationship between the experimental condition, deservingness, and income category. Figure A6 shows the AMCE and AMCE differences with the outcome as the 0-10 deservingness scale. Consistent with the allocation, we find that perceived deservingness is very strongly influenced by income of the recipient, with the tendency stronger for the GST condition. Conversely, COVID-19 relief deservingness is relatively less influenced by pre-crisis income. Additionally, employment status AMCE differences are present where COVID-19 recipients are deemed more deserving if they are under or unemployed.

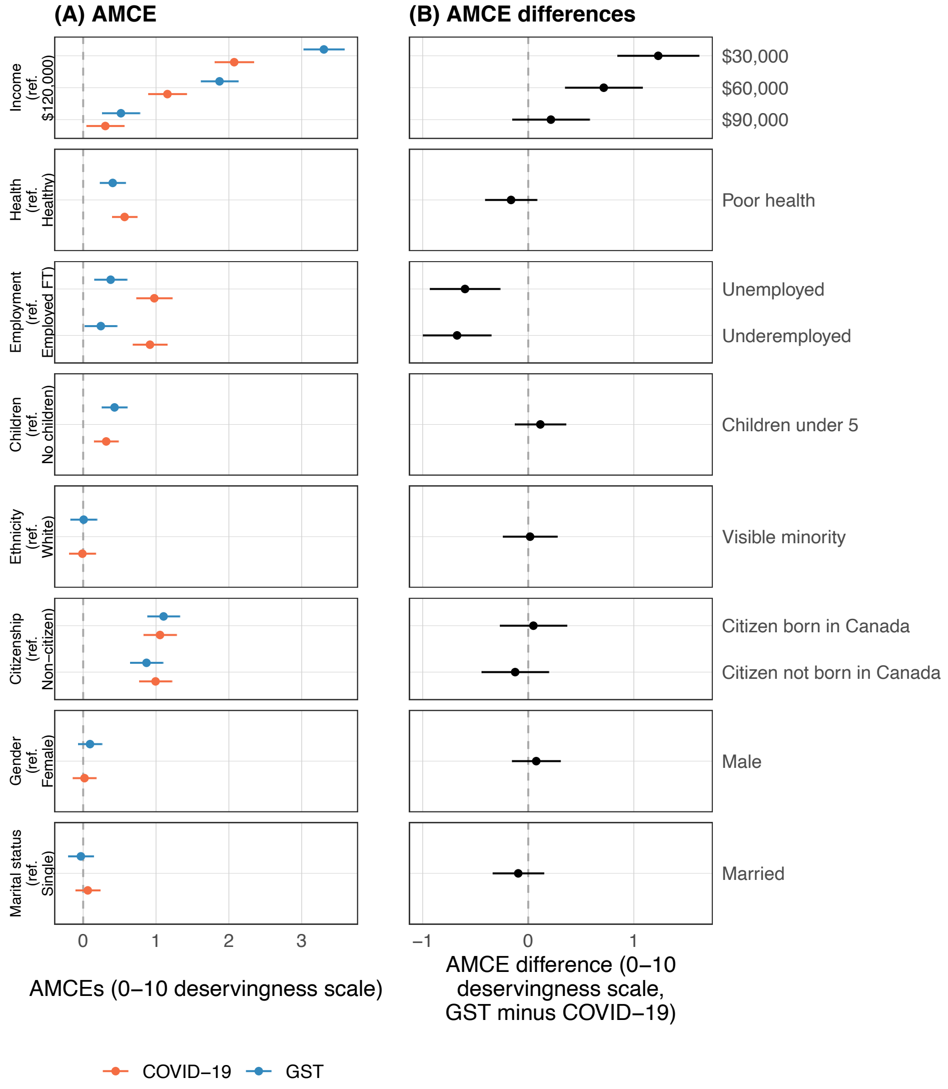


Figure A6: Results for deservingness AMCEs for COVID-19 and GST conditions (panel A), and deservingness AMCE differences (panel B)

Table A7 shows three models which provide additional insight into the underlying dynamics. Model 1 shows the extent to which respondents differ in their evaluations of deservingness based on whether they received the COVID-19 or GST prompt. With random assignment to the condition, we can be confident that respondents consider hypothetical recipients more deserving of COVID-19 assistance as compared to the more general GST rebate. Model 2 again uses deservingness as the dependent variable and looks at the extent to which hypothetical income factors into the decision. Importantly, deservingness increases both for those with lower incomes and this increase is larger for those assigned the GST condition. Model 3 extends Model 3 from Table 2 in the main paper by adding a triple interaction term. This allows us to see whether it is low income recipients that drive the . Consistent with the findings from Model 2, we do find that both deservingness and allocation increases for the GST condition are the result of the $30,000 income category. Conversely, recipient income is less likely to structure deservingness or allocation evaluations for hypothetical COVID-19 aid recipients. This again highlights the finding of a regressive universal redistributive regime associated with the pandemic.

Table A7: Non-registered tests on the relationship between deservingness, income, and the GST condition

| | 1: Deservingness | 2: Income | 3: Allocation |
|---|---|---|---|
| Constant | 5.27 (0.05)* | 5.49 (1.09)* | −0.20 (0.42) |
| Recipient income (ref $120,000) | | | |
| $30,000 | | 2.27 (0.11)* | 0.15 (0.10) |
| $60,000 | | 1.22 (0.11)* | 0.05 (0.08) |
| $90,000 | | 0.40 (0.11)* | −0.01 (0.07) |
| GST condition (ref $120,000) | | | |
| GST | −0.53 (0.07)* | −2.79 (1.53) | −0.26 (0.59) |
| GST x $30,000 | | 1.25 (0.15)* | −0.17 (0.13) |
| GST x $60,000 | | 0.76 (0.15)* | −0.09 (0.11) |
| GST x $90,000 | | 0.22 (0.16) | −0.11 (0.10) |
| Deservingness (ref $120,000) | | | |
| Deservingness | | | 0.18 (0.01)* |
| GST x Deservingness | | | −0.02 (0.02) |
| Deservingness x $30,000 | | | −0.02 (0.02) |
| Deservingness x $60,000 | | | −0.01 (0.01) |
| Deservingness x $90,000 | | | 0.01 (0.01) |
| Triple interaction (ref $120,000) | | | |
| GST x Deservingness x $30,000 | | | 0.07 (0.02)* |
| GST x Deservingness x $60,000 | | | 0.04 (0.02) |
| GST x Deservingness x $90,000 | | | 0.02 (0.02) |
| $R^2$ | 0.01 | 0.66 | 0.78 |
| Adj. $R^2$ | 0.01 | 0.54 | 0.70 |
| Num. obs. | 8551 | 8551 | 8551 |

*$p < 0.01$. Linear regressions, with individual respondent controls and clustered standard errors in parentheses (for Models 2 and 3). Dependent variable for Models 1 and 2 is subjective evaluation of deservingness. Dependent variable for Model 3 is mean-normalized allocation to hypothetical individuals, either under the COVID-19 or GST conditions.
